# Hypocaloric Diet Initiated Post-Ischemia Provides Long-Term Neuroprotection and Promotes Peri-Infarct Brain Remodeling by Regulating Metabolic and Survival-Promoting Proteins

**DOI:** 10.1007/s12035-020-02207-7

**Published:** 2020-11-17

**Authors:** Tayana Silva de Carvalho, Eduardo H. Sanchez-Mendoza, Adriana R. Schultz Moreira, Luiza M. Nascentes Melo, Chen Wang, Maryam Sardari, Nina Hagemann, Thorsten R. Doeppner, Christoph Kleinschnitz, Dirk M. Hermann

**Affiliations:** 1 Department of Neurology, University Hospital Essen, Hufelandstraße 55, D-45122 Essen, Germany; 2 Department of Neurology, University Medicine Göttingen, Göttingen, Germany

**Keywords:** Calorie restriction, Ischemic stroke, Neurological recovery, Neuroprotection, Sirtuin-1

## Abstract

Calorie restriction confers post-ischemic neuroprotection, when administered in a defined time window before ischemic stroke. How a hypocaloric diet influences stroke recovery when initiated after stroke has not been investigated. Male C57BL6/j mice were exposed to transient intraluminal middle cerebral artery occlusion. Immediately post-ischemia, mice were randomized to two groups receiving moderately hypocaloric (2286 kcal/kg food) or normocaloric (3518 kcal/kg) diets ad libitum. Animals were sacrificed at 3 or 56 days post-ischemia (dpi). Besides increased low density lipoprotein at 3 days and reduced alanine aminotransferase and increased urea at 56 days, no alterations of plasma markers were found in ischemic mice on hypocaloric diet. Body weight mildly decreased over 56 dpi by 7.4%. Hypocaloric diet reduced infarct volume in the acute stroke phase at 3 dpi and decreased brain atrophy, increased neuronal survival and brain capillary density in peri-infarct striatum and reduced motor coordination impairment in tight rope tests in the post-acute stroke phase over up to 56 dpi. The abundance of brain-derived neurotrophic factor, the NAD-dependent deacetylase and longevity protein sirtuin-1, the anti-oxidant glutathione peroxidase-3, and the ammonium detoxifier glutamine synthetase in the peri-infarct brain tissue was increased by hypocaloric diet. This study shows that a moderately hypocaloric diet that is initiated after stroke confers long-term neuroprotection and promotes peri-infarct brain remodeling.

**Supplementary Information:**

The online version contains supplementary material available at 10.1007/s12035-020-02207-7.

## Introduction

Caloric restriction extends lifespan, as previously shown in a large number of species that include yeast, nematodes, fruit-flies, mice, and non-human primates [1–3]. In humans, the observations from animals recently prompted the randomized controlled Comprehensive Assessment of the Long-term Effects of Reducing Intake of Energy (CALERIE) trial, in which non-obese adults were randomized to normocaloric diet or a targeted 25% caloric restriction [4]. In the CALERIE trial, caloric restriction induced a reduction of energy expenditure by ~10%, as revealed by the doubly labeled water method, and a reduction of lipid peroxidation, as evaluated by urinary F2-isoprostane measurement [4]. These data demonstrated that sustained metabolic adaptation to the hypocaloric diet is associated with reduced oxidative stress in humans.

Tissue energy requirements and oxidative stress are important factors influencing ischemic injury, neurological recovery, and brain remodeling. Experimental studies in mice and rats using models of focal cerebral ischemia by middle cerebral artery occlusion (MCAO) showed that caloric restriction or fasting prior to a stroke reduced post-ischemic neurological deficits, infarct volume, and brain inflammatory responses [3, 5–9]. In models of global cerebral ischemia induced by bilateral common carotid artery occlusion in rats and gerbils, caloric restriction initiated after ischemia did not influence neurological recovery and neuronal survival but increased acute-phase responses and disturbed terminal axonal and synaptic plasticity [10, 11]. In focal cerebral ischemia, the effects of post-ischemic caloric restriction on brain injury and neurological recovery have not yet been examined, although this question is clinically more relevant than pre-ischemic caloric restriction. To address this issue, we herein exposed mice to intraluminal MCAO and evaluated the effects of a controlled hypocaloric diet (35% less kcal/kg food) that was initiated immediately after stroke on ischemic injury, neurological recovery, and brain remodeling over up to 56 days.

## Materials and Methods

### Animal Ethics, Statistical Planning and Randomization, and Experimenter Blinding

Experiments were approved by local government authorities (Bezirksregierung Düsseldorf) in accordance with E.U. guidelines (Directive 2010/63/EU) for the care and use of laboratory animals. Sample size calculations determined that 12 animals per group were required for the neurological examinations and histochemical studies, given that the effect size was 1.167, the alpha error was 5%, and the beta error (1–statistical power) was 20%. Experimenters were blinded by a third person not involved in the assessments, who randomized the animals in two groups as described below, labeled them as A and B, and provided food pellets as required.

### Food Modifications and Animal Groups

**Fig. 1:**
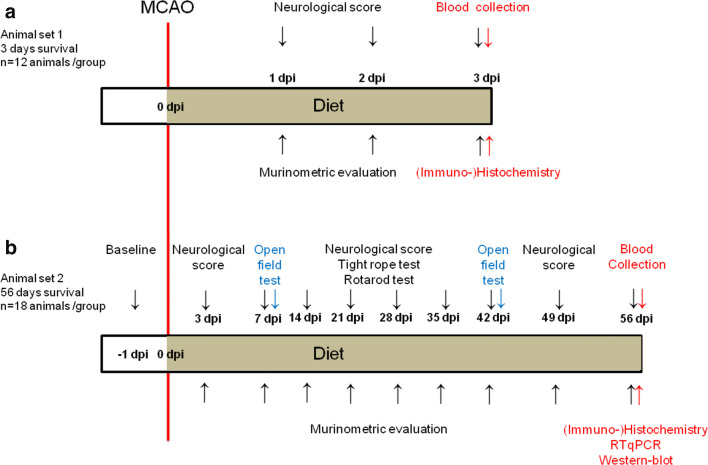
**Experimental procedures and animal groups.** Mice submitted to transient middle cerebral artery occlusion (MCAO) were fed with normocaloric and hypocaloric diets starting immediately after reperfusion over **a** up to 3 days (animal set 1) or **b** up to 56 days (animal set 2). In animal set 1 (in **a**), mice were used for nutritional/murinometric analyses, clinical chemical measurements in peripheral blood, neurological score, histochemistry, and immunohistochemistry (*n* = 12 animals/group). In animal set 2 (in **b**), mice were used for nutritional/murinometric analyses, clinical chemical measurements in peripheral blood, behavioral tests (which comprised neurological score, tight rope, Rotarod and open field tests), histochemistry, immunohistochemistry, and molecular biological studies (real-time quantitative polymerase chain reaction [RTqPCR], Western blots) (*n* = 18) animals/group, of which 12 animals/group were attributed for behavioral tests, nutritional/murinometric analyses, clinical chemical measurements, histochemistry, and immunohistochemistry and 6 animals/group were attributed for molecular biological studies

Adult male C57BL6/J mice (8 weeks, 26-30 g; Harlan-Netherlands, Rossdorf, Germany) exposed to 30 min intraluminal MCAO were randomized to two fixed formula diets provided by an animal nutrition manufacturer: (a) normocaloric nutrition (C1000; 3518 kcal/kg food, 20% protein, 13% lipids; Altromin, Lage, Germany) or (b) hypocaloric nutrition (C1001 mod.; 2286 kcal/kg food, 20% protein, 13% lipids; Altromin), which were administered ad libitum over up to 56 days starting with the induction of intraluminal MCAO. Hence, the hypocaloric diet had 35% less kcal/kg food than the normocaloric diet, which from a nutritional perspective aimed to induce a state of metabolic moderation and frugality without inducing malnutrition syndrome. Post-ischemia, rodents respond sensitively to reduce calorie supply. In gerbils or rats, the reduction of daily calorie supply by 30 or 40% using a fixed food amount protocol (restriction of food pellet access) results in a weight loss of ~30% over 2–3 months [10, 12]. Considering that this weight reduction is probably too severe to induce beneficial effects on stroke recovery, we decided to provide the calorie-reduced diet ad libitum to mice, which allowed the animals to partially compensate for the reduced calorie content by increased food consumption. This dietary protocol resembles a protocol previously used for studying effects of pre-ischemic malnutrition on ischemic brain injury [9], albeit a more severe calorie reduction (1313 kcal/ kg food) was imposed in this earlier study. Throughout the study, animals were housed in groups of 4 animals in group cages (Green line IVC Sealsafe Plus Mouse; Tecniplast, Hohenpeißenberg, Germany) in a 12 h:12 h light/dark cycle. The timeline of experiments is given in Fig. 1. Two sets of experiments were performed, in which animals survived (**A**) 3 days post-ischemia (dpi; *n* = 12 animals/ group) or (**B**) 56 dpi (*n* = 18 animals/ group). Mice sacrificed at 3 dpi were used for nutritional/murinometric analyses, clinical chemical measurements in peripheral blood, neurological score, and histochemistry/immunohistochemistry (*n* = 12 animals/group). Mice sacrificed at 56 dpi were divided into two subsets that were used for (a) nutritional/murinometric analyses, clinical chemical measurements in peripheral blood, behavioral tests and histochemistry/immunohistochemistry (*n* = 12 animals/group) and (b) murinometric analyses and molecular biological studies (real-time quantitative polymerase chain reaction (RTqPCR), Western blots) (*n* = 6 animals/group). For animals sacrificed at 3 dpi and 56 dpi, additional non-ischemic control groups were prepared, which were used for nutritional/murinometric analyses and clinical chemical measurements (*n* = 12 animals/group). These mice were littermates of mice exposed to MCAO, which were randomly selected by the person responsible for animal randomization and blinding.

### Intraluminal MCAO

Mice were anesthetized with 1.0–1.5% isoflurane (30% O_2_, remainder N_2_O). Rectal temperature was maintained between 36.5 and 37.0 °C using a feedback-controlled heating system. Cerebral laser Doppler flow (LDF) was recorded using a flexible probe (Perimed, Järfälla, Sweden) attached to the skull overlying the core of the middle cerebral artery territory. A midline neck incision was made. The left common and external carotid arteries were isolated and ligated, and the internal carotid artery was temporarily clipped. A silicon-coated nylon monofilament (0.21 mm tip diameter; Doccol, Sharon, MA, U.S.A.) was introduced through a small incision of the common carotid artery and advanced to the circle of Willis for MCAO [13]. Reperfusion was initiated 30 min thereafter by monofilament removal. Following surgical interventions, wounds were carefully sutured and anesthesia was discontinued. Non-ischemic mice were not exposed to anesthesia or surgeries. These mice were kept on normocaloric diet for 56 days. Food consumption and calorie intake were measured daily. Body (i.e., nose–anus) length was determined before MCAO. Body weight and body mass index were determined daily (animals sacrificed at 3 dpi) or weekly (animals sacrificed at 56 dpi) before and after MCAO (Fig. 1) [14].

### Neurological Score

At 24 h post-ischemia, 2 dpi (in mice sacrificed at 3 dpi), 3 dpi, 7 dpi, and at weekly intervals thereafter, mice were evaluated using Clark’s neurological score [15], which captures general and focal neurological deficits.

### Tight Rope Test

The tight rope test consists of a 60-cm-long rope that is attached to two opposing platforms. Animals are placed on the middle of the rope and the time until reaching one of the platforms is determined (maximum testing time 60 s) [16, 17]. Animals were trained three times on three consecutive days before MCAO. After a baseline examination, animals were tested weekly until 42 dpi, as shown in Fig. 1. The tight rope test was performed three times at each time point. Means were calculated for each time point. Our group has previously used this protocol for evaluating the efficacy of restorative stroke therapies [16, 17].

### Rotarod Test

The Rotarod is a motor coordination test, which consists of a rotating drum (Ugo Basile, model 47,600, Comerio, Italy), in which animals are placed while the drum is accelerating. The time until each animal drops off the drum is measured (maximum testing time 300 s) [16, 17]. Animals were trained three times each on three consecutive days before MCAO. Following a baseline evaluation, animals were tested weekly until 42 dpi, as shown in Fig. 1. The test was performed three times at each time point. Means were calculated for each time point. Our group has previously used this protocol for evaluating the efficacy of restorative stroke therapies [16, 17].

### Open Field Test

The open field arena is a square platform (52 x 52 x 30 cm) subdivided into one center (31.2 × 31.2 cm), four border (each 10.4 × 31.2 cm), and four corner (each 10.4 × 10.4 cm) fields, in which animals are placed in the center and observed for 300 s for evaluating spontaneous motor behavior [16, 17]. The number of field entries, duration in each field ,and speed were tracked using VideoMot software (version 7.0.1; TSE Systems, Bad Homburg, Germany). The open field test was performed once at baseline, 7 dpi and 42 dpi, as shown in Fig. 1. Our group has previously used this protocol in restorative stroke studies [16, 17].

### Plasma Measurements and Animal Sacrifice

Seventy-two hours or 56 days after MCAO animals were re-anesthetized. In animals sacrificed at 3 dpi and at 56 dpi and their non-ischemic control animals, plasma samples were obtained from the animals’ hearts by cardiac puncture after 5 h fasting that were used for analysis of urea, bilirubin, aspartate aminotransferase (AST), alanine aminotransferase (ALT), total protein, albumin, cholesterol, low-density lipoprotein (LDL), triglycerides, and glucose levels (ADVIA® 2400; Siemens, Erlangen, Germany). Twelve animals per group were transcardially perfused with 4% paraformaldehyde (PFA) in 0.1 M phosphate-buffered saline (PBS). Brains were weighted, post-fixed overnight in 4% PFA in 0.1 M PBS, and cryoprotected by immersion in 30% sucrose in 0.1 M PBS. Brains were frozen and cut into 20-μm-thick coronal cryostat sections that were used for conventional histochemistry. Additional six ischemic animals per group sacrificed at 56 dpi were transcardially perfused with normal saline. These animals were used for Western blots and RTqPCR [9].

### Infarct Volume, Brain Edema, and Brain Volume

Coronal 20 μm sections collected at millimeter intervals across the brain were stained with cresyl violet. Sections were scanned and quantified using Image J software (National Institute of Health, Bethesda, MD, U.S.A.). In animals sacrificed at 3 dpi, infarct volume was measured by subtracting areas of healthy tissue of the ischemic hemisphere from those of the contralesional hemisphere [9, 16]. In animals sacrificed at 56 dpi, striatum volume and whole brain volume were evaluated by analyzing ipsilesional and contralesional brain areas across the forebrain, of which percent volume ratios were determined [16]. At this time point, the infarct is completely resolved. Therefore injury measurements require volume measurements of survival brain tissue [16]. Corpus callosum thickness was evaluated at the bregma level by tracing the corpus callosum area in the ischemic hemisphere from the midline up to one millimeter lateral to the midline [18]. Thereby, the mean corpus callosum thickness was determined.

### Immunohistochemistry of IgG Extravasation

Twenty μm sections obtained from the rostrocaudal level of the midstriatum were rinsed for 20 min in 0.3% H_2_O_2_ in 70% methanol in 0.1 M PBS, immersed in 0.1 M PBS containing 5% bovine serum albumin (BSA) (05470; Sigma-Aldrich, Darmstadt, Germany), and incubated for 1 h in biotinylated anti-mouse IgG (1:100; Santa Cruz, Heidelberg, Germany), followed by diaminobenzidinetetrahydrochloride (DAB) (D5905; Sigma-Aldrich, Darmstadt, Germany) staining with an avidin-biotin complex peroxidase kit (Vectastain Elite; Vector Labs, Burlingame, CA, U.S.A.). IgG extravasation was analyzed by evaluating the brain area exhibiting IgG extravasation [9].

### Immunohistochemistry

Adjacent sections from the same level were immersed in 0.1 M PBS containing 0.1% Triton X-100 (PBS-T) and 10% normal donkey serum (D9663; Sigma-Aldrich). Sections were incubated overnight at 4 °C in monoclonal rat anti-CD45 (1:200; 550,539; BD Biosciences, Heidelberg, Germany), monoclonal rabbit anti-NeuN (1:400; ab177487; Abcam, Cambridge, U.K.), monoclonal rat anti-CD31 (1:500; ab56299; Abcam), polyclonal rabbit anti-ionized calcium binding adaptor protein (Iba)-1 (1:500; Wako Chemicals, Neuss, Germany), monoclonal rat anti-glial fibrillary acidic protein (GFAP) (1:200; 130,300; Invitrogen, Dublin, Ireland), polyclonal rabbit anti-glutamine synthetase (1:250; G2781; BD Sigma-Aldrich), or polyclonal sheep anti-brain-derived neurotrophic factor (BDNF) (1:200; ab75040; Abcam) antibodies that were detected with Cy3, Alexa Fluor-488, Alexa Fluor-550, or Alexa Fluor-594-labeled secondary antibodies (NeuN, CD31, Iba1, GFAP, glutamine synthetase, BDNF), as appropriate, or biotinylated secondary antibodies followed by DAB staining with the avidin-biotin complex peroxidase kit (Vectastain Elite) (CD45). NeuN, Iba1, GFAP, glutamine synthetase, and BDNF labelings were counterstained with Hoechst-33,342 (H1399; Thermo Fisher Scientific, Waltham, MA, U.S.A). Sections were evaluated under a Zeiss AxioObserver.Z1 inverted epifluorescence microscope equipped with Apotome optical sectioning by counting the total number of immunopositive cells or microvessels in the ischemic striatum (CD45, NeuN, CD31, Iba1, GFAP/glutamine synthetase, BDNF) or analyzing the area covered by immunopositive cells (Iba1, GFAP). The latter analysis was preferred to cell countings, since individual cells could not always unequivocally be discriminated. Optical sectioning was used for correction of cell/capillary overcounts [16].

### RTqPCR

From tissue samples harvested from the ischemic middle cerebral artery territory and liver, messenger RNA (mRNA) was extracted using the RNeasy Mini kit (Qiagen, Hilden, Germany). mRNA was converted to cDNA using the high-capacity RNA-to-cDNA kit (Thermo Fisher Scientific). RTqPCR was performed using a StepOnePlus real-time PCR instrument (Thermo Fisher Scientific) with primers designed or selected by the PubMed primer BLAST tool (https://blast.ncbi.nlm.nih.gov/) (Suppl. Table 1). Melting curves were used to confirm the efficiency of the primers. β-glucuronidase (βGluc) was used as housekeeping gene, brain and liver tissue from healthy mice served as control. Results were quantified using the 2-∆∆Ct method [19]. RTqPCR were performed in triplicates, of which mean values were computed for each animal.

### Western Blots

From the same samples, protein samples were collected after 1-bromo-3-chloropropane (B9673; Sigma-Aldrich, Darmstadt, Germany) separation. Ethanol was added and samples centrifuged at 12.000 g for 5 min. This procedure was repeated twice. The resulting pellet was suspended in 4% sodium dodecyl sulfate (SDS) (436,143; Sigma-Aldrich). Protein content was measured using the Bradford method (#500–0113; Bio-Rad, Hercules, CA, U.S.A.). Equal amounts of protein (20 μg) were loaded on 10% SDS-polyacrylamide gels, submitted to SDS-polyacrylamide gel electrophoresis (PAGE), and transferred onto polyvinylidene fluoride (PVDF) membranes (Bio-Rad). Membranes were blocked by 5% nonfat-dried milk (M7409; Sigma-Aldrich) in 50 Mm Tris-buffered saline (TBS) containing 0.1% Tween (P9416; Sigma-Aldrich) for 1 h at room temperature and washed and incubated overnight at 4 °C with monoclonal rabbit anti-sirtuin-1 (Sirt1; 1:2000; ab32441; Abcam), polyclonal rabbit anti-glutathione peroxidase-3 (Gpx3; 1:2000; ab59524; Abcam), polyclonal rabbit anti 4-hydroxy-2-nonenal (4HNE; 1:1000; 393,207, Calbiochem), and polyclonal rabbit anti-β-actin (1:10000; 4967; Cell Signaling, Frankfurt, Germany) antibody. The next day, membranes were washed and incubated with secondary donkey anti-rabbit or anti-goat antibody. Blots were revealed using a chemiluminescence kit (RPN2232; ECL Prime Western Blotting Detection reagents; Amersham, Vienna, Austria) and scanned using a myECL Imager (Thermo Fisher Scientific). Sirt1, Gpx3, and 4HNE abundance was densitometrically evaluated in three independent experiments. The relative abundance of Sirt1, Gpx3, and 4HNE was normalized to protein loading as determined in β-actin blots [9].

### Statistics

Statistical analyses were performed using SPSS for Windows. Murinometric data, nutritional data, LDF recordings, and neurological deficits were analyzed by repeated measurement ANOVA followed by Bonferroni tests as post hoc tests. Plasma measurements, histochemical data, Western blots, and RTqPCR data were analyzed by one-way ANOVA followed by Bonferroni tests or t-tests, as adequate. Plasma measurements, RTqPCR data, and data involving repeated measurements are presented as mean ± S.D. values, all other data as median (mean) ± interquartile ranges (IQR) with minimum and maximum data as whiskers. *P* values < 0.05 were defined to indicate statistical significance.

## Results

### Hypocaloric Diet Induces Mild Body Weight Reduction in Mice Exposed to Focal Cerebral Ischemia

Intraluminal MCAO induced highly reproducible ischemias, as revealed by cerebral LDF measurements (Suppl. Fig. 1a). LDF reproducibly decreased to ~15–20% of baseline values during MCAO, followed by the restitution to baseline values within 20 min after reperfusion (Suppl. Fig. 1a). LDF values, during and after MCAO, did not differ between the two ischemic groups (Suppl. Fig. 1a).

Focal cerebral ischemia was followed by a ~ 13% drop of body weight (Suppl. Fig. 1B) and body mass index (from 0.078 ± 0.006 g/cm^2^ to 0.068 ± 0.005 g/cm^2^ in normocaloric mice). This weight reduction progressively recovered within 56 dpi, when compared with non-ischemic mice. In the first 28 dpi, mice exposed to normocaloric and hypocaloric diets displayed very similar body weight recovery (Suppl. Fig. 1b). Thereafter, mice on the hypocaloric diet exhibited slightly attenuated body weight gains (Suppl. Fig. 1b). After 56 days, the body weight of ischemic mice on hypocaloric diet was 2.0 ± 0.1 g (7.4 ± 0.5%) below ischemic mice on normocaloric diet (Suppl. Fig. 1b). Body weight recovery significantly differed between hypocaloric and normocaloric ischemic animals (diet–time interaction effect in repeated measurement ANOVA: F(1,34) = 2.748, *p* = 0.004).

At 1 dpi, the total amount of food ingested sharply decreased in ischemic compared with non-ischemic mice (from 2.1 ± 0.1 to 1.0 ± 0.7 g/day in ischemic mice on normocaloric diet; from 2.1 ± 0.5 to 0.7 ± 0.4 g/day in ischemic mice on hypocaloric diet; Suppl. Fig. 1c), as did calorie intake (from 7.4 ± 0.4 to 3.7 ± 1.9 kcal/day in ischemic mice on normocaloric diet; from 7.3 ± 1.7 to 1.7 ± 0.9 kcal/day in ischemic mice on hypocaloric diet; Suppl. Fig. 1D). Food and calorie intake transiently increased above baseline levels at 21–35 dpi in ischemic mice on normocaloric diet (Suppl. Fig. 1c, d). Calorie intake was significantly lower throughout the observation period in ischemic mice on hypocaloric diet than ischemic mice on normocaloric diet (main effect of diet in repeated measurement ANOVA: F(1,22) = 38.430, *p* < 0.001; Suppl. Fig. 1d). Total calorie intake over 56 dpi of ischemic mice on hypocaloric diet was 306.5 ± 85.4 kcal, compared with 581.3 ± 68.9 kcal in ischemic mice on normocaloric diet. Hence, total calorie intake in ischemic mice was reduced by hypocaloric diet by 47.3%.

### Hypocaloric Diet Induces Subtle Metabolic Changes in Peripheral Blood

Plasma urea, a marker of protein metabolism [20], and plasma triglycerides, a marker of lipogenesis [21], were significantly elevated in ischemic mice at 56 dpi (Table 1). Plasma urea was higher in ischemic mice on hypocaloric diet than ischemic mice on normocaloric diet (*p* = 0.009; Table 1). Plasma triglycerides did not differ between the two ischemic groups (*p* = 0.362; Table 1). ALT, a marker of liver enzymatic activity [22], was significantly lower in ischemic mice on hypocaloric diet than ischemic mice on normocaloric diet (*p* = 0.013; Table 1). Plasma bilirubin, AST, total protein, albumin, cholesterol, LDL, and glucose did not differ between groups (Table 1). Plasma LDL, a marker of lipogenesis [21], was significantly elevated in ischemic mice at 3 dpi (Suppl. Table 2). Plasma LDL was higher in ischemic mice on hypocaloric diet than ischemic mice on normocaloric diet (*p* = 0.004; Suppl. Table 2).

**Table 1:** Clinical chemical changes in peripheral blood induced by hypocaloric diet

Groups	Urea/ mg/dl	Bilirubin/ mg/dl	AST/ U/l	ALT/ U/l	Protein/ g/dl	Albumin/ g/dl	Cholesterol/ mg/dl	LDL/ mg/dl	Triglycerides/ mg/dl	Glucose/ mg/dl
Non-ischemic normocaloric diet	13.3 ± 3.1	1.3 ± 0.5	247.1 ± 97.2	77.2 ± 44.8	3.9 ± 0.6	2.8 ± 0.5	124.6 ± 23.7	8.4 ± 2.9	60.4 ± 13.3	188.0 ± 36.9
Ischemic normocaloric diet	16.9 ± 6.0	1.6 ± 1.0	290.7 ± 188.8	82.7 ± 36.2	4.4 ± 1.3	3.2 ± 1.6	132.1 ± 44.9	11.7 ± 5.9	85.3 ± 49.6 ^**^	187.7 ± 40.0
Ischemic hypocaloric diet	18.3 ± 3.7 * ^##^	1.3 ± 0.4	298.3 ± 133.4	50.9 ± 16.2 * ^#^	4.5 ± 1.2	2.9 ± 1.9	121.6 ± 41.9	8.8 ± 2.1	110.8 ± 62.9 *	171.6 ± 32.1

Data are means ± S.D. values, evaluated at 56 dpi. **p < 0.01/ *p < 0.05 compared with ischemic mice on normocaloric diet, ^##^*p* < 0.01/ ^#^*p* < 0.05 compared with non-ischemic mice on normocaloric diet (*n* = 12 animals/ group). *ALT* alanine aminotransferase, *AST* aspartate aminotransferase, *LDL* low-density lipoprotein

### Hypocaloric Diet Does Not Influence Neurological Deficits in the Acute Stroke Phase but Enhances Motor Coordination Performance in the Post-Acute Stroke Phase

**Fig. 2:**
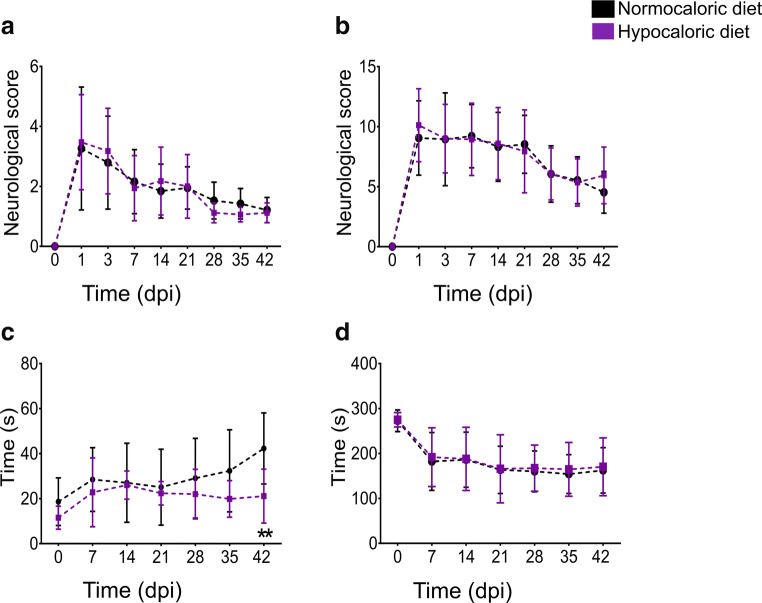
**Hypocaloric diet does not influence neurological deficits in the acute stroke phase, but enhances motor-coordination performance in the tight rope test in the post-acute stroke phase. a** General and **b** focal neurological deficits examined by Clark’s neurological score and **c, d** motor-balance deficits evaluated by tight rope and Rotarod tests in mice exposed to intraluminal MCAO, which received a normocaloric or hypocaloric diet for 56 days. Data are means ± S.D. values. **p < 0.01 compared with ischemic mice on normocaloric diet (*n* = 10 animals/ group)

**Fig. 3:**
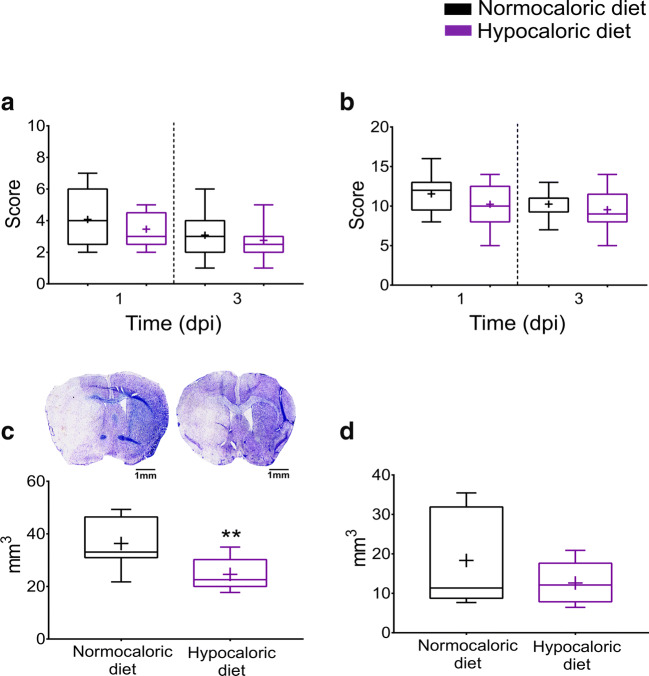
**Hypocaloric diet protects against ischemic injury in the acute stroke phase. a** General neurological deficits and **b** focal neurological deficits evaluated by the Clark score, **c** infarct volume, and **d** brain edema outlined on cresyl violet-stained brain sections of mice exposed to intraluminal MCAO, which received a normocaloric or hypocaloric diet for 3 days. Representative photographs are shown. Data are medians (lines inside boxes)/means (crosses inside boxes) ± interquartile ranges (IQR; boxes) with minimum/maximum values as whiskers. **p < 0.01 compared with ischemic mice on normocaloric diet (*n* = 12 animals/ group). Scale bar, 1 mm (in **(C)**

Focal cerebral ischemia induced reproducible general and focal neurological deficits, as revealed by Clark’s neurological score, tight rope, and Rotarod tests (Fig. 2a–d**,** Fig. 3a, b). These deficits decreased with time but remained detectable until the end of this study. The hypocaloric diet did not induce any changes of general (Fig. 2a**,** Fig. 3a) and focal (Fig. 2B**,** Fig. 3B) neurological deficits, as evaluated by Clark’s neurological score, which sensitively measures neurological impairment in the early stroke recovery phase (main effect of diet in repeated measurement ANOVA for general deficits: F(1,18) = 0.003, *p* = 0.955; main effect of diet in repeated measurement ANOVA for focal deficits: F(1,18) = 0.135, *p* = 0.715). Interestingly, the hypocaloric diet significantly reduced motor coordination deficits in the tight rope test at 42 dpi (main effect of diet in repeated measurement ANOVA: F(1,18) = 4.864, *p* = 0.041; Fig. 2c). Motor coordination performance in the Rotarod test was not influenced by the hypocaloric diet (main effect of diet in repeated measurement ANOVA: F(1,18) = 0.127, *p* = 0.725; Fig. 2d). Spontaneous motor activity, that is, animal speed in the open field test, and exploration behavior, evaluated by the time in the center of the open field test, did not differ between groups (Suppl. Fig. 2a, b). None of the animals on hypocaloric diet exhibited signs of undernutrition, such as exsiccation or fur abnormalities. None of the animals revealed stool paleness or increased stool sample size, which are symptoms indicative of intestinal malabsorption syndrome [9].

### Hypocaloric Diet Reduces Ischemic Injury in the Acute Stroke Phase

**Fig. 4:**
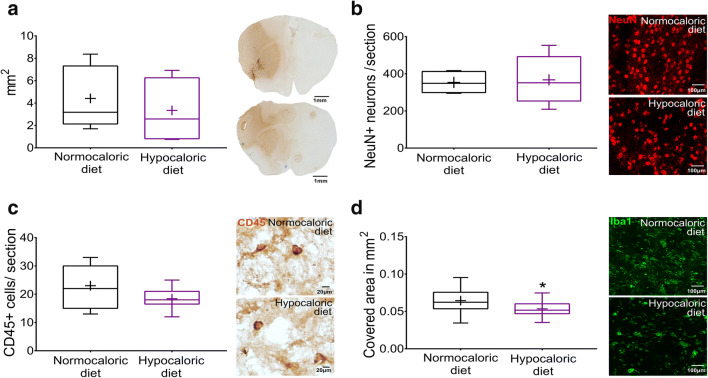
**Hypocaloric diet reduces microglial abundance in the ischemic brain tissue, but does not influence brain leukocyte infiltration in the acute stroke phase. a** Serum IgG extravasation evaluated by immunohistochemistry, **b** neuronal number in the ischemic striatum assessed by NeuN immunohistochemistry, **c** brain leukocyte infiltration in the ischemic striatum examined by CD45 immunohistochemistry, and **d** microglial abundance in the ischemic striatum evaluated by Iba1 immunohistochemistry in mice exposed to intraluminal MCAO, which received a normocaloric or hypocaloric diet for 3 days. Representative microphotographs are shown. Data are medians (lines inside boxes)/means (crosses inside boxes) ± interquartile ranges (IQR; boxes) with minimum/maximum values as whiskers. *p < 0.05 compared with ischemic mice on normocaloric diet (*n* = 12 animals/ group). Scale bars, 1 mm (in **a**)/100 μm (in **b**, **d** )/ 20 μm (in **c**)

Histochemical studies showed that the hypocaloric diet significantly reduced infarct volume (*p* = 0.035; Fig. 3c), but did not influence brain edema or serum IgG extravasation, which is a marker of blood-brain barrier permeability, in the acute stroke phase at 3 dpi (*p* = 0.339 and *p* = 0.563, respectively; Fig. 3d**,** Fig. 4a). The number of surviving neurons and number of brain-infiltrating leukocytes examined by NeuN and CD45 immunohistochemistry were unchanged (*p* = 0.809 and *p* = 0.105, respectively; Fig. 4b, c), whereas microglial abundance evaluated by Iba1 immunohistochemistry was significantly reduced by the hypocaloric diet in the peri-infarct striatum (*p* = 0.047; Fig. 4d).

### Hypocaloric Diet Prevents Brain Atrophy and Promotes Peri-Infarct Brain Remodeling in the Post-Acute Stroke Phase

**Fig. 5:**
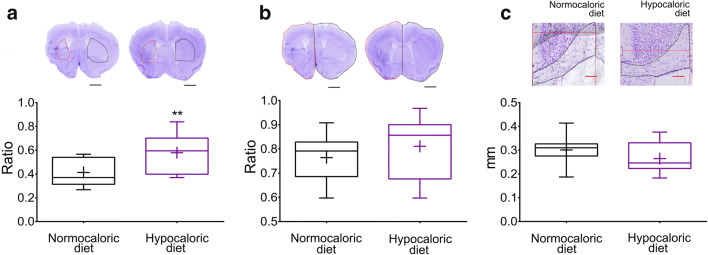
**Hypocaloric diet protects the brain against striatal atrophy in the post-acute stroke phase. a** Striatum volume, **b** whole brain volume, and **c** corpus callosum thickness evaluated on cresyl violet stained brain sections in mice exposed to intraluminal MCAO, which received a normocaloric or hypocaloric diet for 56 days. Representative photographs are shown. Data are medians (lines inside boxes)/means (crosses inside boxes) ± interquartile ranges (IQR; boxes) with minimum/maximum values as whiskers. **p < 0.01 compared with ischemic mice on normocaloric diet (*n* = 12 animals/group). Scale bars, 1 mm (in **a, b**)/100 μm (in **c**)

**Fig. 6:**
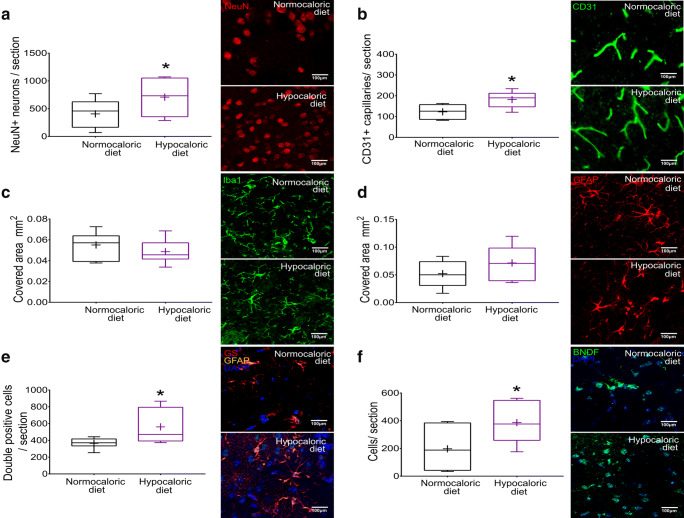
**Hypocaloric diet induces long-term neuroprotection and increases brain capillary density. a** Neuronal survival in the peri-infarct striatum assessed by NeuN immunohistochemistry, **b** brain capillary density in the peri-infarct striatum examined by CD31 immunohistochemistry, **c** microglial abundance in the peri-infarct striatum evaluated by Iba1 immunohistochemistry, **d** astroglial reactivity in the peri-infarct striatum assessed by GFAP immunohistochemistry, **e** glutamine synthetase expression in GFAP+ astrocytes examined by GFAP/ glutamine synthetase double immunohistochemistry, and **f** BDNF expression in the peri-infarct striatum evaluated by immunohistochemistry in mice exposed to intraluminal MCAO, which received a normocaloric or hypocaloric diet for 56 days. Representative microphotographs are shown. Data are medians (lines inside boxes)/means (crosses inside boxes) ± interquartile ranges (IQR; boxes) with minimum/maximum values as whiskers. *p < 0.05 compared with ischemic mice on normocaloric diet (*n* = 12 animals/ group). Scale bars, 100 μm (in **a–f**)

Subsequent studies showed that the hypocaloric diet significantly reduced the loss of striatum volume (*p* = 0.005; Fig. 5a) in the post-acute stroke phase, i.e., at 56 dpi, but did not influence whole brain volume (*p* = 0.410; Fig. 5b) or corpus callosum thickness (*p* = 0.273; Fig. 5c). At 56 dpi, the brain infarct is fully resolved. Neuroprotection translates into the prevention of brain atrophy. The number of surviving neurons (*p* = 0.048; Fig. 6a) and brain capillaries (*p* = 0.042; Fig. 6b) were significantly increased in the peri-infarct striatum by the hypocaloric diet, as revealed by NeuN and CD31 immunohistochemistry, whereas microglial abundance (*p* = 0.295; Fig. 6c) and astroglial scar formation (*p* = 0.198; Fig. 6d) examined by Iba1 and GFAP immunohistochemistry were unchanged. Interestingly, the number of glutamine synthetase+/GFAP + astrocytes was significantly increased in the peri-infarct striatum of mice on hypocaloric diet (*p* = 0.026; Fig. 6e). Glutamine synthetase catalyzes the formation of the amino acid glutamine by transferring otherwise toxic ammonium to glutamate under conditions of proteolysis [23]. Glutamine synthetase thus protects the brain in catabolic states. The number of BDNF+ cells in the peri-infarct striatum was significantly increased by the hypocaloric diet (*p* = 0.044; Fig. 6f).

### Hypocaloric Diet Increases the NAD-Dependent Deacetylase Sirtuin-1 and the Anti-Oxidant Glutathione Peroxidase-3 in the Peri-Infarct Brain and Liver

**Fig. 7:**
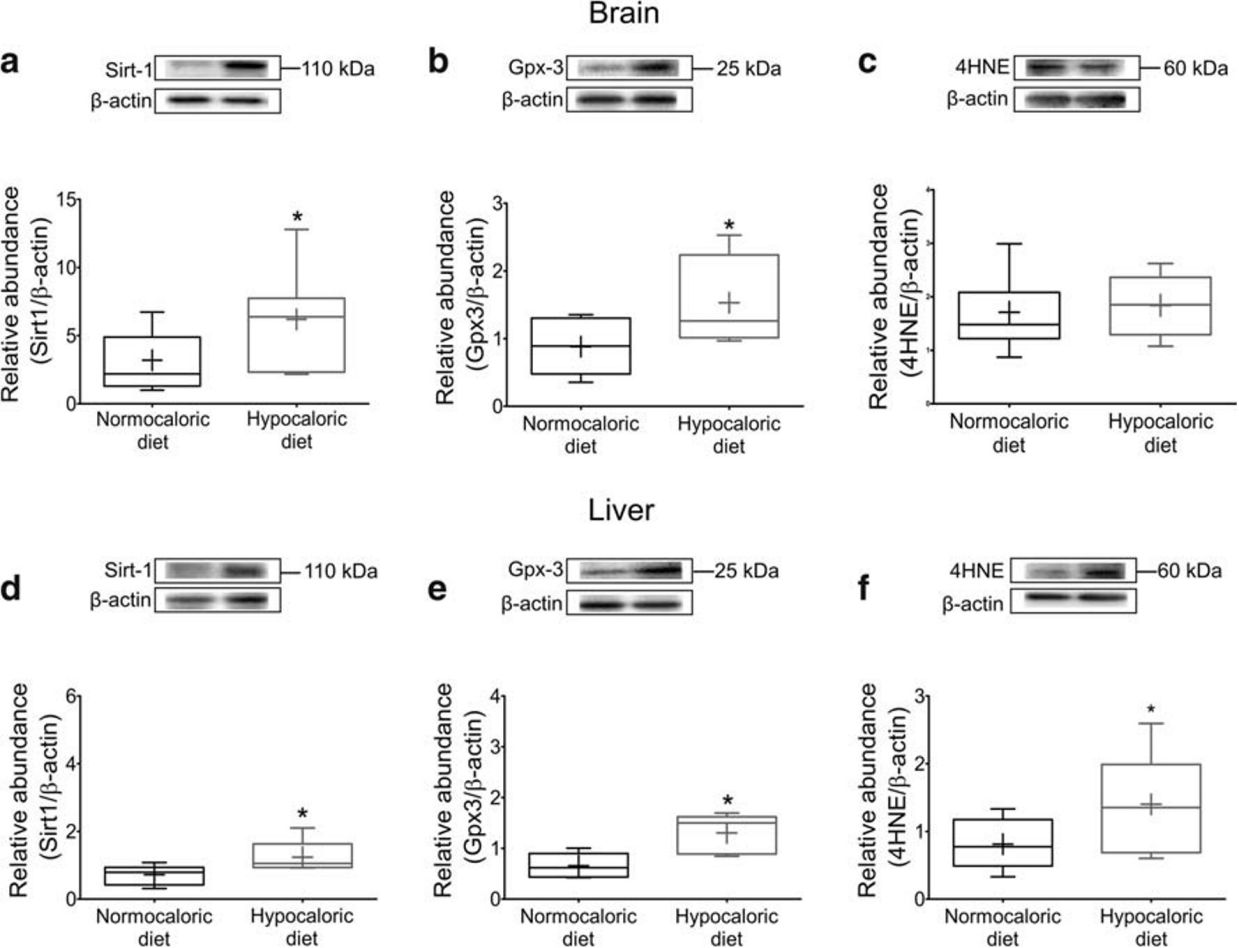
**Hypocaloric diet increases protein abundance of sirtuin-1 and glutathione peroxidase-3 in the peri-infarct brain and liver.** Western blot analysis of **a, d** sirtuin-1 (Sirt1), **b, e** glutathione peroxidase-3 (Gpx3), and **c, f** the reactive aldehyde 4-hydroxy-2-nonenal in the ischemic brain (in **a–c**) and the liver (in **d–f**) of mice exposed to intraluminal MCAO, which received a normocaloric or hypocaloric diet for 56 days. Representative Western blots are also shown. Data are medians (lines inside boxes)/means (crosses inside boxes) ± interquartile ranges (IQR; boxes) with minimum/maximum values as whiskers. *p < 0.05 compared with ischemic mice on normocaloric diet (*n* = 6 animals/group)

Whole brain weight was not influenced by hypocaloric nutrition (0.39 ± 0.03 g in ischemic mice on normocaloric diet, 0.36 ± 0.04 g in ischemic mice on hypocaloric diet; *p* = 0.095), whereas liver weight was significantly reduced in mice on hypocaloric diet (1.4 ± 0.2 in ischemic mice on normocaloric diet, 1.1 ± 0.2 g in ischemic mice on hypocaloric diet; *p* = 0.003). Western blots revealed that the abundance of Sirt1 and Gpx3 proteins in the peri-infarct brain (p = 0.026 and *p* = 0.013, respectively; Fig. 7a, B) and liver (*p* = 0.027 and *p* = 0.036, respectively; Fig. 7d, e) was significantly increased by the hypocaloric diet. The level of 4-HNE, which is a product of lipid peroxidation that affects mitochondrial function by impairing ATPase activity [24], was not altered by the hypocaloric diet in the ischemic brain (*p* = 0.178; Fig. 7c) but increased by the hypocaloric diet in the liver (*p* = 0.034; Fig. 7f).

RTqPCR showed that the hypocaloric diet did not elevate *Sirt1* and *Gpx3* expression on the mRNA level in the brain (Suppl. Table 2) and liver (Suppl. Table 3), suggesting that the protein changes noted were a consequence of a reduced protein degradation rather than increased de novo expression of the genes. Likewise, mRNAs for the growth factor *insulin-like growth factor-1* (*Igf1*), which has insulin-like properties [25], the pro-inflammatory cytokine *interleukin-1β* (*Il1β*) and *superoxide dismutase-1* (*Sod1*), which degrades superoxide anions to hydrogen peroxide [26], were unchanged in both tissues. mRNAs for the transcription factor *nuclear factor-κb* (*Nfκb*) and the dismutase *Sod2* were not altered in the liver.

## Discussion

Using a moderately hypocaloric diet, which we administered to mice for up to 56 days after intraluminal MCAO, we herein show that hypocaloric diet reduces infarct volume, promotes post-ischemic motor-coordination recovery, increases long-term neuronal survival, increases brain capillary density, and reduces brain atrophy in the peri-infarct striatum, which was the brain region most strongly affected by the stroke. Body weight changes induced by the hypocaloric diet were mild, and only subtle metabolic changes, i.e., increased LDL at 3dpi and reduced alanine aminotransferase and increased urea at 56 days, were noted in the blood of mice on hypocaloric diet. On the molecular level, an increased abundance of the growth factor BDNF, the NAD-dependent deacetylase and longevity protein Sirt1, the anti-oxidant Gpx3, and the ammonium consuming glutamine synthetase were observed in the peri-infarct brain tissue of mice on hypocaloric diet at 56 dpi. Our results suggest that a moderately hypocaloric diet that is initiated after stroke confers sustained neuroprotection and promotes peri-infarct brain remodeling.

To the best of our knowledge, no studies so far examined the effects of a post-ischemically administered hypocaloric diet on neurological deficits and ischemic injury. Focal cerebral ischemia studies in mice and rats showed that caloric restriction or fasting prior to a stroke reduced neurological deficits, infarct volume, and brain inflammatory responses, when animals were subsequently exposed to MCAO [3, 5–8]. In these studies, caloric restriction was achieved by reducing the animals’ access to food pellets by 40 [3, 5, 8] or 30% [6] for up to 8 weeks. Fasting was induced by completely preventing food access for 3 days [7]. Neurological deficits and brain injury were evaluated at 24 h post-MCAO [3, 5, 8], in two studies up to 7 dpi [7] or 14 dpi [6]. Two studies evaluated the effects of post-ischemic caloric restriction (30% calorie reduction or combined protein-energy restriction) on neurological deficits and ischemic injury after global cerebral ischemia in gerbils or rats [10, 11]. In these studies, caloric restriction initiated after global cerebral ischemia did not influence neurological recovery and neuronal survival but increased acute-phase responses and disturbed terminal axonal and synaptic plasticity [10, 11].

The restriction of food pellets reduces the animals’ access to essential food ingredients, micronutrients, and vitamins. It may therefore not be a desirable strategy in stroke patients from a nutritional point of view. To circumvent this problem, we herein exposed mice ad libitum to a balanced diet containing 35% less calories, which had identical lipid, protein, micronutrient, and vitamin content as the normocaloric diet. Interestingly, mice on hypocaloric diet consumed lower amounts of food (in grams) in the first days after stroke than mice on normocaloric diet. Despite lower food intake (in grams and calories), the animals were protected against ischemic injury, indicated by reduced infarct volume at 3 dpi. In ischemic mice on hypocaloric diet, total calorie intake over 56 dpi was reduced by 47.3% compared with ischemic normocaloric mice. This significant calorie reduction resulted in a mild decrease of body weight by 2.0 ± 0.1 g (7.4%) at 56 dpi, and nutritional status was not compromised. Besides moderately elevated plasma urea, a marker of protein metabolism [20], and moderately reduced ALT, a marker of hepatic enzymatic activity [22], no additional plasma changes of metabolic markers were found. Our data suggest that metabolic needs had adapted to the reduced energy supply. A single study so far examined the effects of a hypocaloric diet that was administered ad libitum to mice over 7–30 days before MCAO [9]. In this diet, calorie content was reduced by as much as 63%. During diet exposure, body weight decreased by ~ 20% over 30 days [9]. The nutritional status of mice on this more severely hypocaloric diet was significantly compromised. The mice exhibited an intestinal malabsorption syndrome with pale stool, enlarged stool samples, and blood beddings on stool [9]. In this earlier study, hypocaloric diet exposure for 14 days reduced neurological deficits, infarct volume, neuronal injury, and blood-brain barrier breakdown at 24 h post-MCAO, whereas prolonged hypocaloric diet exposure for 30 days failed to show neuroprotective effects probably due to compromised post-ischemic reperfusion resulting from progressive animal exhaustion [9]. The degree of calorie reduction explains different findings in this earlier and the present study.

Animals on hypocaloric diet revealed a signature of survival-promoting, metabolism-related, and anti-oxidant responses in the brain and liver, which likely contributed to stroke outcome. In the peri-infarct brain tissue, the pleiotropic growth factor BDNF and the NAD-dependent deacetylase and longevity protein Sirt1, which stabilizes mitochondrial function and metabolism partly by deacetylating the transcription regulator peroxisome proliferator-activated receptor-γ coactivator-1α (PGC1α) [27], were elevated by hypocaloric diet at 56 dpi, as was Gpx3, a peroxidase degrading hydrogen peroxide [28], and glutamine synthetase, which catalyzes the formation of the amino acid glutamine by transferring otherwise toxic ammonium to glutamate under conditions of proteolysis [23]. Following pre-ischemic exposure to hypocaloric diet, the increased abundance of Sirt1 protein and increased expression of *Sirt1* and *Gpx3* mRNAs had already been reported in ischemic brain and liver [9]. In the present study, Sirt1 and Gpx3 proteins, but not their mRNAs were increased in the liver of mice on hypocaloric diet, indicating adaptation of liver metabolic state to lower energy supply. Notably, 4-HNE, which is a product of lipid peroxidation that affects mitochondrial function by impairing ATPase activity [24], was unchanged in the brain but increased in the liver of mice on hypocaloric diet. This observation suggests that the utilization of energy stores under conditions of a hypocaloric diet may result in oxidative stress that carefully needs to be considered in dietary interventions. Strengths of this study are the well-controlled nutritional assessments and the use of a broad battery of behavioral tests, structural volumetry/planimetry studies, immunohistochemical, and molecular biological analyses. We performed this study on young, male mice without vascular risk factors or comorbidities, because no information was hitherto available about the consequences of calorie reduction post-stroke on ischemic injury and neurological recovery. Studies on aged mice, mice with vascular risk factors (e.g., hyperlipidemia or obesity) or comorbidities (e.g., diabetes), and female mice should be conducted in the future. Studies on the consequences of different types of dietary modifications (including protein-reduced diets), their dose-response relationships, timing, and underlying mechanisms should also be considered. These studies might provide clinically meaningful insights in how dietary modifications influence ischemic brain injury and post-ischemic brain remodeling in different stroke settings.

## Supplementary Information

ESM 1 (PDF 705 kb).

## Data Availability

The raw data supporting the conclusions of this manuscript will be made available by the authors, without undue reservation, to any qualified researcher.
