# Supplementary material for: Hypocaloric Diet Initiated Post-Ischemia Provides Long-Term Neuroprotection and Promotes Peri-Infarct Brain Remodeling by Regulating Metabolic and Survival-Promoting Proteins
Source: Mol Neurobiol. 2020 Nov 17;58(4):1491–503. doi: 10.1007/s12035-020-02207-7 (PMC7932971; doi:10.1007/s12035-020-02207-7)
Supplement: Supplementary file 1 — (PDF 705 kb). [file 12035_2020_2207_MOESM1_ESM.pdf]

Hypocaloric diet initiated post-ischemia provides long-term neuroprotection and promotes peri-infarct brain remodeling by regulating metabolic and survival-promoting proteins

Tayana Silva de Carvalho, PhD; Eduardo H. Sanchez-Mendoza, PhD; Adriana R. Schultz Moreira, PhD; Luiza M. Nascentes Melo, MSc; Chen Wang, MSc; Maryam Sardari, PhD; Nina Hagemann, PhD; Thorsten R. Doeppner, MD; Christoph Kleinschnitz, MD; Dirk M. Hermann, MD

Departments of Neurology, [1]University Hospital Essen, Essen, and [2]University Medicine Göttingen, Göttingen, Germany

Running title: Hypocaloric diet promotes peri-infarct brain remodeling

Supplemental figures: 2

Supplemental tables: 4

Correspondence: Prof. Dirk M. Hermann, MD
Department of Neurology, University Hospital Essen
Hufelandstr. 55, D-45122 Essen, Germany
Phone: +49-201-723-2814, Fax: +49-201-723-5534
E-mail: dirk.hermann@uk-essen.de
Orcid: https://orcid.org/0000-0003-0198-3152

Supplemental Figures and Tables:

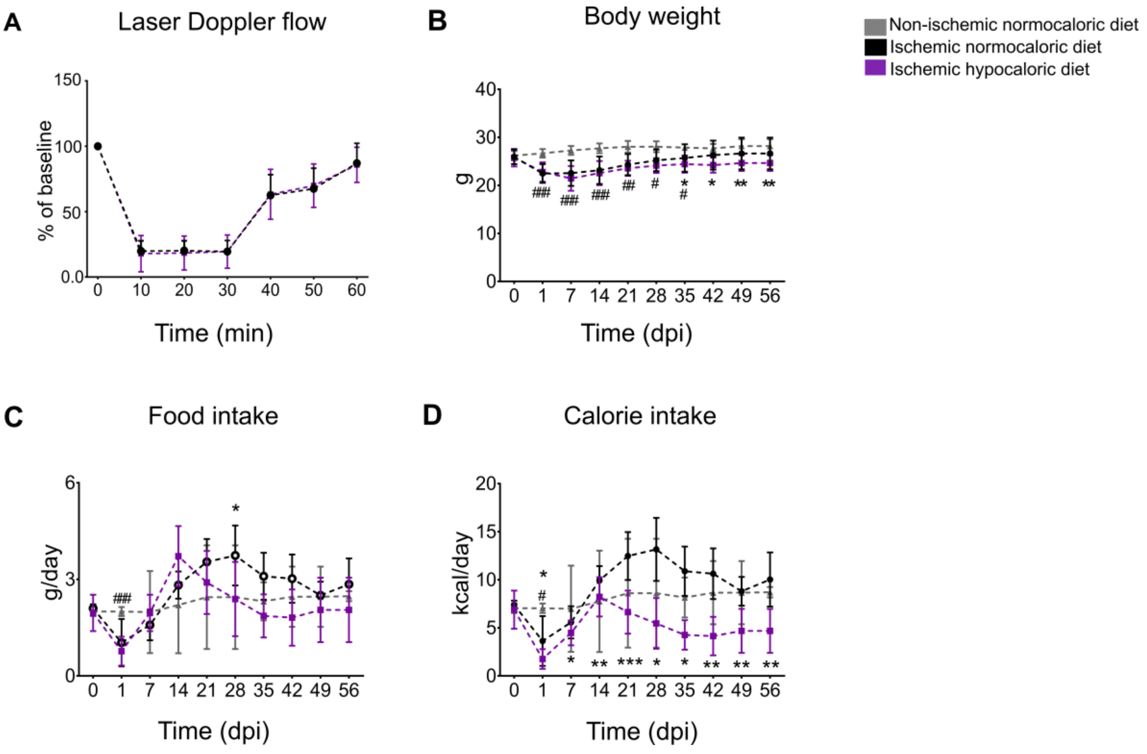

Supplemental Figure 1. Long-term hypocaloric diet induces mild body weight reduction in mice exposed to focal cerebral ischemia. (A) Laser Doppler flow (LDF) recordings above the core of the middle cerebral artery territory, **(B)** body weight, **(C)** daily food intake and **(D)** daily calorie intake of mice exposed to intraluminal middle cerebral artery occlusion (MCAO), which were fed *ad libitum* with a normocaloric diet (3518 kcal/kg) or a hypocaloric diet (2286 kcal/kg) for 56 days. Data are means ± S.D. values. $^{***}p<0.001$/ $^{**}p<0.01$/ $^{*}p<0.05$ compared with ischemic mice on normocaloric diet/ $^{\#\#}p<0.01$/ $^{\#}p<0.05$ compared with non-ischemic mice on normocaloric diet (n=18 [in **(A)**]/ n=12-18 [in **(B)**]/ n=12 [in **(C)** and **(D)**] animals/ group).

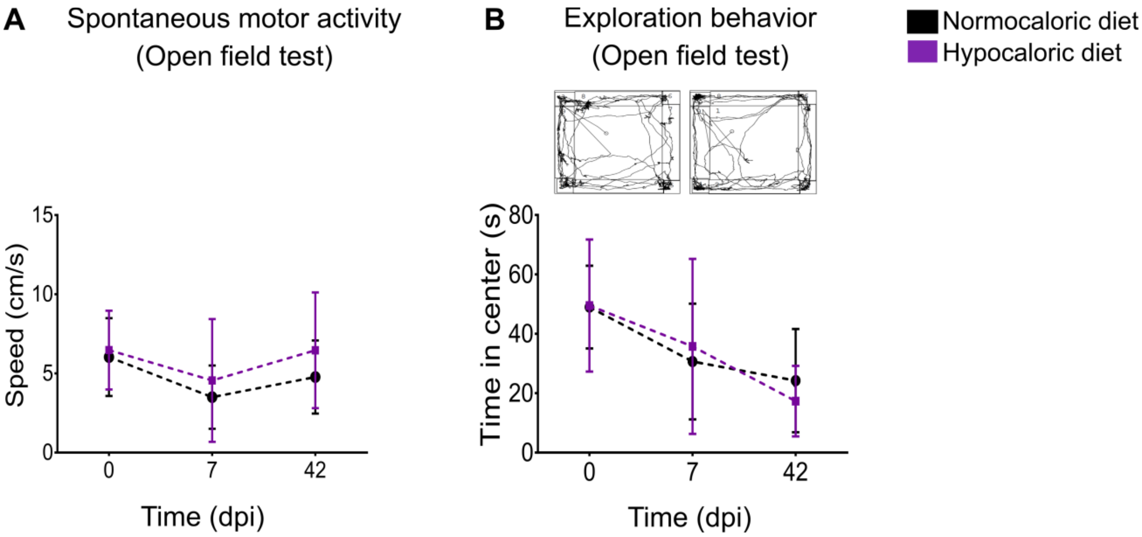

Supplemental Figure 2. Hypocaloric diet does not influence spontaneous motor activity and exploration behavior. (A) Spontaneous motor activity, i.e., mean speed in the open field test, and **(B)** exploration behavior, i.e., time in the center in the open field test, of mice exposed to intraluminal middle cerebral artery occlusion (MCAO), which received a normocaloric or hypocaloric diet for 56 days. Data are means ± S.D. values. No significant group differences were noted (n=10 animals/ group).

Supplemental Table 1. List of PCR primers

Primer	Sequence (5'->3')		Tm	G-C (%)	Gene bank number
Sirt1	Forward	GATGACAGAACGTCACACGC	59.56	55.00	NM_019812.3
	Reverse	ATTGTTCGAGGATCGGTGCC	60.46	55.00	
Igf1	Forward	GACTCAGAAGTCCCCGTCCC	61.61	65.00	NM_010512.5
	Reverse	GCATTTTCTGCTCCGTGGG	59.49	57.89	
Il1β	Forward	TCTTTGAAGTTGACGGACCCC	60.20	52.38	NC_000068.7
	Reverse	CTTGTTGATGTGCTGCTGCG	60.73	55.00	
Nfκb	Forward	TTTCGACTACGCAGTGACGG	60.39	55.00	NM_008689.2
	Reverse	GCTAAGTGTAAGACACTGTCCC	58.41	50.00	
Sod1	Forward	CATCCACTTCGAGCAGAAGGC	61.34	57.14	NM_011434.1
	Reverse	GGTACAGCCTTGTGTATTGTCCC	61.18	52.17	
Sod2	Forward	GAACAACAGGCCTTATTCCGC	61.32	60.00	NM_013671.3
	Reverse	GTGTATCTTTCAGTAACATTCTCCC	59.31	50.00	
Gpx3	Forward	GCACTACAAGAAGAACTTGGGC	59.77	50.00	NM_001329860.1
	Reverse	TCGAACATACTTGAGACTGGGG	59.50	50.00	
βGluc	Forward	TGGTATAAGACGCATCAGAAGCC	60.49	47.83	NC_000071.6
	Reverse	GGTACTCCTCACTGAACATGCG	60.99	54.55	

Sirt1, sirtuin-1; Igf1, insulin-like growth factor-1; Il1β, interleukin-1β; Nfκb, nuclear factor-κb; Sod1, superoxide dismutase-1; Sod2, superoxide dismutase-2; Gpx3, glutathione peroxidase-3; βGluc, β-glucuronidase.

Supplemental Table 2. Clinical chemical changes in peripheral blood induced by hypocaloric diet at 3 days post-stroke

Groups	Urea/ mg/dl	Bilirubin/ mg/dl	AST/ U/l	ALT/ U/l	Total Protein/ g/dl	Albumin/ g/dl	Cholesterol/ mg/dl	LDL/ mg/dl	Triglycerides/ mg/dl	Glucose/ mg/dl
Non-ischemic normocaloric diet	24.2 ± 4.5	0.4 ± 0.0	121.2 ± 44.0	36.0 ± 8.7	4.7 ± 0.1	3.1 ± 0.0	123.8 ± 9.7	7.4 ± 1.2	81.8 ± 16.8	203.6 ± 34.1
Ischemic normocaloric diet	18.9 ± 6.7	0.8 ± 0.2*	206.3 ± 73.5*	48.3 ± 32.4	4.5 ± 0.5	3.0 ± 0.3	129.6 ± 23.2	12.7 ± 4.2*	63.3 ± 30.9**	148.3 ± 32.1**
Ischemic hypocaloric diet	16.4 ± 5.26*	0.73 ± 0.4	155.2 ± 65.4	31.2 ± 14.0	4.6 ± 0.3	3.0 ± 0.2	132.2 ± 17.0	18.2 ± 4.6*** ##	52.8 ± 9.8**	170.6 ± 20.1*

Data are means ± S.D. values, evaluated at 3 dpi. ***$p<0.001$/ **$p<0.01$/ *$p<0.05$ compared with ischemic mice on normocaloric diet, ##$p<0.01$ compared with non-ischemic mice on normocaloric diet (n=12 animals/ group). ALT, alanine aminotransferase; AST, aspartate aminotransferase; LDL, low-density lipoprotein.

Supplemental Table 3. Expression of metabolism-related, inflammatory and anti-oxidant genes in the brains of ischemic mice exposed to normocaloric or hypocaloric diet

Groups	***Sirt1***	***Igf1***	***Il1β***	***Sod1***	***Gpx3***
Normocaloric Diet	3.42 ± 1.04	1.51 ± 0.50	2.44 ± 0.57	2.00 ± 0.37	3.19 ± 0.41
Hypocaloric diet	2.54 ± 0.59	1.26 ± 0.08	1.901 ± 0.43	1.83 ± 0.28	2.03 ± 0.71*

Data are fold changes, expressed as mean ± S.D., evaluated at 56 days post-ischemia (dpi). *$p<0.05$ compared with ischemic mice on normocaloric diet (n=6 mice/ group). Sirt1, sirtuin-1; Igf1, insulin-like growth factor-1; Il1β, interleukin-1β; Sod1, superoxide dismutase-1; Gpx3, glutathione peroxidase-3.

Supplemental Table 4. Expression of metabolism-related, inflammatory and anti-oxidant genes in the liver of ischemic mice exposed to normocaloric or hypocaloric diet

Groups	***Sirt1***	***Igf1***	***Nfκb***	***Sod1***	***Sod2***	***Gpx3***
Normocaloric Diet	1.51 ± 0.37	1.15 ± 0.24	0.99 ± 0.25	1.12 ± 0.23	1.11 ± 0.23	1.21 ± 0.32
Hypocaloric diet	2.07 ± 0.64	1.36 ± 0.36	0.99 ± 0.32	1.00 ± 0.10	0.89 ± 0.18	0.89 ± 0.17*

Data are fold changes, expressed as mean ± S.D., evaluated at 56 dpi.*p<0.05 compared with ischemic mice on normocaloric diet (n=6 mice/ group). Sirt1, sirtuin-1; Igf1, insulin-like growth factor-1; Nfκb, nuclear factor-κb; Sod1, superoxide dismutase-1; Sod2, superoxide dismutase-2; Gpx3, glutathione peroxidase-3.
